# Access to insurance navigation support through the State Health Insurance Assistance Program (SHIP)

**DOI:** 10.1093/haschl/qxae072

**Published:** 2024-05-21

**Authors:** Melissa M Garrido, David Biko, Allison Dorneo, Paul R Shafer, Austin B Frakt

**Affiliations:** Partnered Evidence-based Policy Resource Center, Boston VA Healthcare System, Boston, MA 02130, United States; Department of Health Law, Policy & Management, Boston University School of Public Health, Boston, MA 02118, United States; Partnered Evidence-based Policy Resource Center, Boston VA Healthcare System, Boston, MA 02130, United States; Department of Health Law, Policy & Management, Boston University School of Public Health, Boston, MA 02118, United States; Partnered Evidence-based Policy Resource Center, Boston VA Healthcare System, Boston, MA 02130, United States; Department of Health Law, Policy & Management, Boston University School of Public Health, Boston, MA 02118, United States; Partnered Evidence-based Policy Resource Center, Boston VA Healthcare System, Boston, MA 02130, United States; Department of Health Law, Policy & Management, Boston University School of Public Health, Boston, MA 02118, United States; Partnered Evidence-based Policy Resource Center, Boston VA Healthcare System, Boston, MA 02130, United States; Department of Health Law, Policy & Management, Boston University School of Public Health, Boston, MA 02118, United States; Department of Health Policy and Management, Harvard TH Chan School of Public Health, Boston, MA 02115, United States

**Keywords:** Medicare, Medicaid, dual eligible, navigation support, counseling

## Abstract

Medicare enrollment is complex, particularly for low-income individuals who are dually eligible for Medicare and Medicaid, and the wrong plan choice can adversely impact beneficiaries’ out-of-pocket costs and access to providers and medications. The State Health Insurance Assistance Program (SHIP) is a federal program that provides counseling on Medicare coverage, but the degree to which SHIP services are accessible to low-income beneficiaries is unknown. We interviewed SHIP counselors and coordinators to characterize factors affecting access to and quality of SHIP services for low-income beneficiaries. Availability of volunteers was cited as the primary barrier to SHIP services. Topics related to dual eligibility for Medicare and Medicaid were frequently covered in counseling sessions, and staff expressed a desire for more training related to Medicaid and integrated-care programs. Our results suggest that additional counselors and increased training on topics relevant to dually eligible individuals may improve SHIP's ability to provide health insurance–related information to low-income Medicare beneficiaries.

## Teaser

Enrolling in Medicare is becoming increasingly complex. The complexity increases further for low-income individuals who are dually eligible for Medicaid and Medicare. The wrong plan choice can adversely impact beneficiaries’ out-of-pocket costs and access to providers and medications. An information resource for beneficiaries is the State Health Insurance Assistance Program (SHIP), which is overseen by the Administration for Community Living. Recent federal communications tout SHIP as a source of unbiased information that can be used to combat deceptive marketing of Medicare Advantage plans, which often target low-income beneficiaries. However, there are concerns about the degree to which SHIP services are accessible to beneficiaries, particularly those who are low-income.

## Introduction

Enrolling in Medicare is becoming increasingly complex.^1^ The complexity increases further for low-income individuals who are dually eligible for Medicaid and Medicare. The wrong plan choice can adversely impact beneficiaries’ out-of-pocket costs and access to providers and medications.^2^ Medicare provides information on different coverage options via their website, medicare.gov, and a toll-free number. An additional information resource is the State Health Insurance Assistance Program (SHIP), which is overseen by the Administration for Community Living (ACL) and which operates in all 50 states, Washington, DC, Puerto Rico, Guam, and the US Virgin Islands.

Through SHIP, the federal government provides grants to states to support counseling and education on Medicare coverage. Funding is determined by the number of Medicare beneficiaries in the state and the number of low-income and rural beneficiaries in the state.^3,4^ In turn, states subcontract to local agencies, including Area Agencies on Aging (AAAs) and senior centers, that are responsible for the program's day-to-day operations. Regional or local coordinators oversee the efforts of trained certified paid and volunteer counselors, who provide free counseling and education on Medicare coverage to beneficiaries. Recent federal communications tout SHIP as a source of unbiased information that can be used to combat deceptive marketing of Medicare Advantage (MA) plans,^5^ which often target low-income beneficiaries.^6^

However, there are concerns about the degree to which SHIP services are accessible to beneficiaries, particularly those who are low-income. SHIP funding through the Medicare Improvements for Patients and Providers Act is based, in part, on the percentage of low-income beneficiaries who reside in each state.^4^ Especially for beneficiaries living in low-income neighborhoods, SHIP access may be affected by availability of volunteers, transportation, virtual counseling, and services in languages other than English. The degree to which these are available throughout the nation is unknown.

Most available information on SHIP access comes from state-level congressional reports that list metrics about the number of beneficiaries who interact with the program. A recent evaluation of California's SHIP identified needs to improve counselors’ knowledge about integrated-care options and maintain an adequate supply of bilingual volunteers.^7^ Using state web directories of SHIP services, we have previously demonstrated that individuals living in areas without access to an in-person SHIP site are more likely to have low income.^8^ However, little is known about factors affecting access to or quality of SHIP services in areas where services are available, nor about the content of SHIP counseling sessions.

Evidence on low-income individuals’ access to SHIP services and opportunities for improvement in service delivery is needed to guide nationwide efforts to improve access to health insurance–related information. To obtain this evidence, we conducted semi-structured interviews with SHIP staff—counselors and coordinators—from across the United States.

## Data and methods

### Study population

To ensure we captured a broad range of experiences, we recruited participants from a random sample of SHIP locations, stratified by Zip Code Tabulation Area (ZCTA), median household income, urban/rural status, and number of available Medicare plans.^8–10^ We ensured our selections were geographically representative (see the Appendix for more details). This study was deemed exempt by the Boston University Medical Campus Institutional Review Board. Our reporting includes items listed in the Consolidated criteria for REporting Qualitative research (COREQ) checklist.^11^

### Recruitment

Outreach was facilitated by ACL staff—prior to recruitment, we presented on a call to state SHIP directors to tell them about the study. To locate participants, we emailed state SHIP directors in states with our identified sites and asked if they were willing to pass along our study information to staff at identified sites. The ACL staff were copied on emails. State directors then either provided us with contact information for potential participants or forwarded an email describing the project to potential participants. We attempted to reach state directors a maximum of 3 times, and we attempted to contact participants up to 4 times. Additional respondents were identified through snowball sampling. Interviews were conducted between April 2023 and September 2023. We offered respondents a $50 electronic gift card in return for participation.

### Interviews

We developed separate interview guides for coordinators and counselors (see the Appendix). We asked coordinators about recruitment and retention of volunteers and paid staff. We asked counselors about common topics of counseling sessions. We asked all participants about their experiences serving dual eligibles and other low-income populations, including whether individuals sought information in-person or via phone calls, and whether beneficiaries encounter scheduling, transportation, comprehension or language barriers when seeking information from a SHIP. Prior to initiating interviews, we asked ACL staff to review the interview guides and provide feedback about clarity and content. Two doctoral students (D.B., A.D.) conducted interviews via Zoom and audio-recorded and transcribed the interviews.

### Analysis

Three team members (D.B., A.D., M.M.G.) developed a codebook through an iterative process, including both inductive and deductive codes. Codes were based on our interview guide questions and on Penchansky and Thomas’ concept of access, which includes constructs of availability, acceptability, accessibility, and accommodation.^12^ Briefly, availability is the relationship between offered and needed services, and accessibility captures efforts and costs required to reach services. Accommodation refers to the systems involved in scheduling, sorting, and managing appointments for beneficiaries to receive services. Acceptability captures attitudes and preferences of beneficiaries and service providers. Because SHIP services are provided at no charge to beneficiaries, we did not include the remaining construct of affordability. Three team members coded 1 full transcript and excerpts of another to ensure there was consensus on the codebook. The remaining transcripts were double-coded.

We used a framework matrix to organize codes and identify themes, paying particular attention to themes relevant to low-income beneficiaries’ experiences. Analyses were facilitated by Lumivero’s NVivo release 14. Findings are illustrated with representative quotes, lightly edited for readability.

## Results

We received responses from 24 of 28 state directors whom we contacted. Of 36 participants identified by state directors or other participants, we made contact with 25 and 22 agreed to participate. Two others agreed to participate but did not attend the interview; 1 declined because they were too busy. Our sample included 9 counselors and 13 coordinators from 16 unique locations (from 15 states). Participants were balanced in representation of urban vs rural areas, median household income within the ZCTA of the SHIP site, complexity of Medicare options, and state oversight model (Table 1). Interviews lasted a mean of 45 minutes (range: 28–63 minutes). We achieved data saturation (“informational redundancy”^13^) in our interviews.

**Table 1.** Characteristics of interview participants (*n* = 22).

Characteristic	*n* (%)
Participant role^a^	
Coordinator	13 (59%)
Counselor	9 (41%)
Region	
Midwest	8 (36%)
Northeast	4 (18%)
South	4 (18%)
West	6 (27%)
Site located in urban area^b^	10 (45%)
Site located in county with higher than the median number of Medicare plans	9 (41%)
Site located in lower quartile of median household income	9 (41%)
State SHIP program oversight	
Department of Insurance	5 (23%)
Department of Commerce or Economic Security	4 (18%)
Department of Health or Human Services	4 (18%)
Department of Aging	7 (32%)

Abbreviation: SHIP, State Health Insurance Assistance Program.

^a^Some coordinators had both coordinating and counselor roles. We list those individuals as coordinators.

^b^Two counselors joined the sample through snowball sampling; specific location information from these individuals is not available.

### Participant background

A wide range of experience was represented in our sample: coordinators had between 6 months and 17 years of experience and counselors had 7 months to 5 years of experience. Some coordinators had worked for a state or county agency for several years before transitioning into the SHIP role. The SHIP operations vary by state, and coordinators ranged from being responsible for a single metropolitan area to multiple counties. Of the 13 coordinators, 2 had explicit dual counselor/coordinator roles. Others would not regularly provide counseling but would train, recruit, and support counselors in navigating complex topics. Still others were not trained for counseling and therefore would not provide counseling services. Of the 9 counselors, 4 were paid staff members and 5 were volunteers.

### Demographics of clientele

All SHIP staff encountered clients newly enrolling in and already enrolled in Medicare. Most participants reported that low-income or dually eligible individuals make up a large part of their client base.

### Access to SHIP services

#### Availability of SHIP staff

In many sites, volunteers provide most of the counseling. In other sites, coordinators lead counseling activities. Most sites seek volunteers with health, social service, or insurance backgrounds who are willing to make a commitment to complete intensive training. In 1 site, the coordinator noted that current volunteers are not diverse and that they would like to improve the diversity of their volunteer base. In another, the counselor noted their site is staffed mostly by non-Hispanic White counselors and thought this might discourage individuals of other backgrounds from seeking assistance.

Many participants cited difficulties in recruiting volunteers, because the information that volunteers are required to learn is complex. Moreover, volunteers often leave during or near the end of training because they feel overwhelmed by the amount of information they need to know (Table 2, Quote 1).

**Table 2.** Barriers to providing SHIP services include availability of volunteers and complexity of information that counselors must relay.

Quote number	Topic	Quote
1	Availability of volunteers	“… I would not be surprised if you can’t get new volunteers for this job. It is extremely difficult…. We had somebody who wanted to volunteer and he went through the video training, did very well on that, sat in on some sessions, and wound up saying, I can’t do that. And he was an operations manager in the company.” —Counselor ID22
2	Complexity of information	“Medicaid's … a relatively rough subject right now because of the pandemic ending. A lot of my staff and volunteers are really learning about all of this stuff still. So I will usually take on the more complex calls.” —Coordinator ID15

Abbreviation: SHIP, State Health Insurance Assistance Program.

Once volunteers complete training and start providing services, however, retention is improved. Turnover was noted as an issue for paid staff more often than for volunteers. Both volunteers and paid staff noted that they receive satisfaction from helping people, although some paid coordinators mentioned concerns with excessive workload or low pay. Particularly during open enrollment, demand for services is high and beneficiaries may need to be placed on a waiting list to receive SHIP services.

#### Transportation

Transportation was mentioned infrequently as a barrier to seeking SHIP services. When present, participants mentioned that transportation barriers were more likely to occur among low-income beneficiaries living in rural areas with no public transportation, and who did not have a car and had to rely on someone else to drive them to appointments. For beneficiaries with transportation difficulties, phone or virtual appointments were offered as an option if a city or county transportation service was unavailable. In some sites, counselors conduct home visits. However, 1 participant noted that securing transportation was “not within our scope of work” (Coordinator [ID11]).

#### Virtual counseling

The SHIP staff described a mix of virtual (telephone or video conference) and face-to-face service delivery. Most programs went fully virtual during the COVID-19 pandemic but now provide some in-person counseling. Virtual services are used more frequently in rural areas. Caretakers, Medicare pre-enrollees, and individuals with mobility or transportation limitations were mentioned as the most likely to use virtual services. Beneficiaries who prefer in-person services include those without a computer or computer skills and dually eligible individuals with complex plan options.

#### Language

In most sites, services in languages other than English were described. Spanish was the most frequently cited language, but staff in many areas mentioned concentrations of regional immigrant populations seeking services in Somali, Vietnamese, and Russian. Although some sites have multilingual staff, most reported using a phone-based language-line interpretation service to meet communication needs. Overall, experiences with the language-line service were positive, although 1 staff member noted challenges in translating Medicare jargon. One counselor mentioned challenges when she could not understand beneficiaries well, but they declined using an interpreter. Occasionally, beneficiaries would bring a friend or family member to translate. Others noted additional accommodations that are needed for beneficiaries who are blind or deaf, including navigating virtual Zoom services or hiring sign language interpreters.

### Content of SHIP counseling sessions

#### Guiding choices between Traditional Medicare and MA

One of the most commonly discussed topics during counseling sessions is helping newly eligible enrollees understand differences between Traditional Medicare (TM) and MA. Counselors use the medicare.gov plan finder tool to facilitate these conversations, and many ask guided questions to streamline the presented information and provide beneficiaries with a set of options that may best suit their finances and care needs. Counselors ask questions about patterns of health care use and pre-existing conditions, willingness to stay in network, patterns of travel or “snowbird” status, and desire for vision and dental coverage. As they discuss options with clients, common topics include the implications of not signing up for Medigap as a new enrollee, the use of copays in MA plans, and the potential for needing increased premiums to get desired dental or vision coverage through MA. Counselors also help clients evaluate whether their medications and preferred providers are covered under different plans. SHIP staff emphasized that they could only provide information and options, and that the final choice of TM or MA was up to the client.

#### Topics specific to low-income or dually eligible individuals

Medicaid, dual eligibility, and cost concerns are among the most frequently discussed topics in counseling sessions. Low-income subsidies and Medicare Savings Programs are also frequently discussed, although many clients come to counseling sessions unaware of programs’ details. SHIP staff noted the importance of screening individuals for programs, and many noted that screening was part of nearly every counseling encounter (Table 3, Quote 1).

**Table 3.** Many SHIP counseling topics are specific to or relevant to low-income beneficiaries.

Quote number	Topic	Quote
1	Screening individuals for means-tested programs	“Nobody's really come up to me… and said, ‘I'm interested in knowing about special needs plans.’ It's more of that conversation of me initiating it because I can see that they might be eligible for it.” —Counselor ID7
2	Redetermination of Medicaid eligibility for enrollees nearing 65 years	“[A commonly discussed topic is] I've been on Medicaid and I'm aging out of Medicaid. And I don't have a lot of money. And now I have to pay for my insurance?…. What's going on? But it turns out that they're dual-eligible because of their income so they can get both Medicaid and Medicare.” —Coordinator ID11
3	Unwinding of Medicaid benefits due to the end of the public health emergency	“During the pandemic, people that were on Medicaid were not being reviewed to see if they should be taken off. So that is happening now… but many people who would have lost their Medicaid during the pandemic period are just now finding out. So I'm getting a lot of those now.” —Counselor ID22
4	Correcting misconceptions from advertisers, agents, or brokers	“There's a lot of backing them off the ledge, if you will, thanks to those TV commercials now.” —Counselor ID21
5	Untangling unwanted enrollment in Medicare Advantage plans	“…They [brokers or agents] will enroll people over the phone and then we're getting the phone calls like, hmm, I'm not sure I did the right thing. So that's when we bring them in. And fortunately if they've never been in a Medicare Advantage plan before, it's an easy fix to get them out. Unfortunately if they're not eligible for a special enrollment period, for some people, it's been over the year, they have to just kind of bite the bullet.” —Coordinator ID14

Abbreviation: SHIP, State Health Insurance Assistance Program.

A frequently encountered topic was the redetermination of Medicaid eligibility for enrollees nearing 65 years (Table 3, Quote 2). In addition, the unwinding of Medicaid benefits after the end of the COVID-19 public health emergency was frequently mentioned. Staff reported helping clients respond to recertification notices and apply for new benefits when needed to ensure continuous coverage (Table 3, Quote 3).

Other topics that were mentioned less frequently included availability of dual-eligible special needs plans (D-SNPs) and the interaction between Medicaid and MA. One participant noted that the available MA plans in their state were not tailored to dually eligible individuals. Another noted that D-SNPs were being advertised to clients in their area, but most providers were not participating in the plans.

In addition to discussing Medicare coverage, some SHIP staff mentioned receiving questions about assistance with groceries, both in response to Supplemental Nutrition Assistance Program (SNAP) benefits returning to pre–public health emergency levels and in response to television advertisements about grocery benefits.

#### Correcting misconceptions from advertising and insurance brokers and agents

The SHIP staff noted that they frequently receive questions from beneficiaries about plans they have seen advertised on television or that were recommended to them by an insurance agent (who generally represents a single insurer), broker (who works with multiple insurers), or a friend. Advertisements are generally broadly targeted, so beneficiaries learn of plans that may not be available in their county. Misleading advertisements were frequently mentioned; beneficiaries, including those with lower incomes, approach counselors with expectations that plans will give them money back or offer far-ranging coverage without additional costs. Counselors work with beneficiaries to clear up misconceptions and provide education around Medicare fraud and abuse (Table 3, Quote 4).

In most cases, counselors work with clients to ensure they are well prepared for a conversation with a broker or agent and not susceptible to recommendations. However, staff mentioned cases where clients go to a broker or agent to buy a Medigap plan after consulting with a SHIP counselor but then get talked into an MA plan. In other cases, clients seek out SHIP counselors for assistance with disenrolling from an MA plan (Table 3, Quote 5).

In the more complicated cases, clients have signed up for both MA and Medigap in response to misleading advertising, and most counselors work to reverse the enrollment when possible. Some SHIPs work with Senior Medicare Patrol or other fraud-detection lines and report inappropriate agent or broker behavior that has negatively impacted their clients.

### Barriers to providing SHIP services to low-income individuals

Many SHIP staff members expressed a desire to have more training about Medicaid. When asked about topics that were challenging to address, coverage for dually eligible beneficiaries was the most frequently cited topic (Table 2, Quote 2).

Some statements indicated that staff potentially misunderstand some coverage options. For instance, in a state that has D-SNPs available, a participant mentioned that MA was often the best option for dually eligible individuals. In another state, a participant discussed broadened eligibility for the Medicare Savings Program in response to a question about integrated-care models. One participant described learning about MA options from YouTube content created by insurance agents.

Although access to beneficiaries’ Medicaid benefits information was available in some sites, several participants also noted that the provision of services is hampered because they do not have visibility into the Medicaid program—they can only help with applications. Difficulties with providing information to individuals with limited education, or who will not answer the phone for follow-up conversations, or those experiencing homelessness were also mentioned.

## Discussion

Through interviews with SHIP staff from across the United States, we characterized barriers to low-income individuals’ access to SHIP services and opportunities for improvement in service delivery. Availability of volunteers was cited as the primary barrier. Topics related to dual eligibility for Medicare and Medicaid were frequently covered in counseling sessions, and staff expressed a desire for more training related to Medicaid and integrated-care programs.

Recruiting volunteers who are comfortable providing counseling on complex topics is a challenge at many sites. The subject matter is complex for counselors as well as beneficiaries; intensive training is provided but it does not cover all topics encountered by counselors. There is often greater demand for services than availability of volunteers. Other insurance and benefit navigation programs also use volunteers to stretch limited budgets,^14,15^ but it is unclear whether they have similar challenges with recruitment. Although no national directory of SHIP counselors (paid or unpaid) is available, the use of volunteers to provide counseling and other services to Medicare beneficiaries was commonly reported by interview participants. Additional financial support to hire counselors may help SHIPs meet demand for services.

From the perspective of SHIP staff, neither language nor transportation were commonly seen as barriers to SHIP service receipt. Services are often provided by phone or video when in-person visits are not possible. The transportation difficulties that were noted were most likely to occur in low-income and rural individuals, groups that often experience transportation-related barriers to general health-related services.^16,17^ Future work should assess access from the beneficiaries’ perspective.

Our findings support others’ calls for increased resources to facilitate access to integrated-care plans.^7,18,19^ Dually eligible beneficiaries are a large part of the client base for most SHIP sites, and coordination of benefits for dually eligible beneficiaries was described as one of the most complex issues SHIP staff handle. Many SHIP staff noted that more training on helping dually eligible beneficiaries would be useful. In addition, some of the responses to our questions suggested that integrated-care models may not be well understood by all and could be a focus of expanded training offerings. Improved access to individual records from state Medicaid offices may also improve service provision for dually eligible beneficiaries.

Our findings also support federal recommendations to use SHIP to combat misinformation from deceptive marketing.^5^ SHIP staff reported spending a great deal of time responding to questions based on TV ads, suggestions from agents and brokers (who, unlike SHIP counselors, can recommend specific plans and are compensated, in part, from enrolling beneficiaries in MA plans), and friends’ recommendations. They also reported working with beneficiaries to resolve unwanted MA enrollment and report fraudulent brokers or agents. However, future research should evaluate the accuracy and completeness of information provided by counselors to beneficiaries.

### Limitations

Our results should be interpreted in light of several limitations. First, no national directory of SHIP counselors exists, so we relied on state directors to refer participants to us. Therefore, our results may not reflect perspectives of all SHIP staff. We tried to minimize the likelihood that state directors referred their “star” staff by asking for contact information for individuals associated with specific sites. Second, a SHIP site's address may not reflect where counselors provide services, particularly in the sites where we had the address of an AAA and not a local community organization and in the sites that were situated within a multi-county service area.^8^ The breadth of service areas covered by some of our participants precluded a systematic investigation of differences in service provision across lower and higher income areas. Finally, we obtained information on counselors’ views of the experiences of beneficiaries who successfully accessed services, not the experiences of beneficiaries themselves. Future work should examine access from the perspective of beneficiaries who did and did not access services. Despite these limitations, to our knowledge, these data present the most in-depth examination to date into barriers and facilitators of SHIP service provision.

## Conclusion

SHIP works with Medicare beneficiaries, many of whom are low-income, to provide counseling on coverage options. Additional counselors and increased training on topics relevant to dually eligible individuals may improve SHIP's ability to provide health insurance–related information to low-income Medicare beneficiaries and to combat misinformation from deceptive marketing.

## Supplementary Material

qxae072_Supplementary_Data
