# Supplementary material for: Access to insurance navigation support through the State Health Insurance Assistance Program (SHIP)
Source: Health Aff Sch. 2024 May 21;2(6):qxae072. doi: 10.1093/haschl/qxae072 (PMC11192052; doi:10.1093/haschl/qxae072)
Supplement: qxae072_Supplementary_Data [file qxae072_supplementary_data.zip › SHIP Aim 2 appendix May 6 2024.docx]

**Appendix – Stratified sample of SHIP locations and Interview guides**

**Stratified random sample of SHIP locations**

To ensure we captured a broad range of experiences, we recruited participants from a stratified random sample of SHIP locations. First, we identified the Zip Code Tabulation Area (ZCTA) in which the site or associated AAA was located and generated a list of sites in the top and bottom quartiles of median household income. Within quartiles, we stratified sites by urban/rural status (Rural-Urban Community Codes of 1-3 vs 4-9) and complexity of Medicare options (above vs below median number of available Medicare plans [Medicare Advantage, Prescription Drug Plans, Special Needs Plans, and Medicare-Medicaid [dually eligible] plans]).^1^ Site characteristics were obtained from the 2018 Agency for Healthcare Research and Quality Social Determinants of Health file and 2022 CMS landscape files.^2,3^ From each of these 8 strata, we randomly selected two sites. We added four additional sites to increase geographic representation across Census regions, and then ensured that our selections included representatives from state programs run by different departments (e.g., insurance or health and human services), which may have different organizational structures and processes. When we could not recruit a participant from a stratum, we replaced the location with another location from the same stratum.

1. Garrido MM, Dorneo A, Adelberg M, Biko D, Shafer P, Frakt AB. Potential inequities in access to in-person SHIP counseling services. *Am J Manag Care*. 2024;30(2):e46-e51. doi:10.37765/ajmc.2024.89500

2. IMPAQ International. Social Determinants of Health (SDOH) Beta Data Files, Data Source Documentation. Published online November 16, 2020. https://www.ahrq.gov/sdoh/data-analytics/sdoh-data.html

3. Centers for Medicare and Medicaid Services. PDP State/County Penetration 2022/01. Published online 2022. https://www.cms.gov/research-statistics-data-and-systemsstatistics-trends-and-reportsmcradvpartdenroldatapdp-state/pdp-statecounty-penetration-2022-01

**SHIP Coordinator & Counselor Interview Guides**

| **Topic** | **Coordinator** | **Counselors** |
| --- | --- | --- |
| Background | Could you talk a little about your role as a coordinator? | Can you tell me about the activities you complete as a counselor? |
|  | Do you coordinate counselors across more than one site where in-person counseling is offered? |  |
|  | How long have you been in this role? | How long have you been working as a SHIP counselor? |
| Financial and Service Administration | How involved are you with securing or managing funding for your SHIP site/region? |  |
|  | During your time as a coordinator has your SHIP site/region received any funding for SHIP services from any non-federal sources? |  |
|  | In an average month, do your counselors provide more in person or virtual services — including video, phone, or any other form of online consults? | In an average month, do you provide more in person or virtual services — including video, phone, or any other form of online consults? |
|  |  | What elements of service have beneficiaries mentioned as being particularly helpful? |
|  | Do beneficiaries ever mention difficulties scheduling appointments for SHIP counseling? | Do beneficiaries ever mention difficulties scheduling appointments for SHIP counseling? |
|  | Do beneficiaries ever experience difficulties securing transportation for in-person SHIP counseling services? | Do beneficiaries ever experience difficulties securing transportation for in-person SHIP counseling services? |
|  | What languages do your counselors offer counseling services in? | What languages do you offer counseling services in? How about your colleagues? |
|  | Do beneficiaries with limited English proficiency ever experience difficulties in locating a counselor with whom they can communicate? | Do beneficiaries with limited English proficiency ever experience difficulties in locating a counselor with whom they can communicate? |
| **Recruitment and Training** | Do you work primarily with volunteers, paid counselors, or a mix of volunteer and paid staff? |  |
|  | Could you talk about the process you go through to identify and recruit volunteers to your SHIP site? |  |
|  | What difficulties, if any, do you face in recruiting volunteers or paid staff? |  |
|  | What difficulties, if any, do you face in retaining counselors? |  |
|  | What is helpful for recruiting or retaining counselors? |  |
|  |  | How did you find your way to being a counselor? |
|  |  | Could you give a short description of your previous work experiences and educational background? |
|  |  | Were there any formal requirements that you needed to meet in order to be eligible to become a counselor? |
|  | Could you talk a bit about the process for training counselors? | Could you describe the training process for becoming a counselor? |
|  |  | Have you received specific training regarding integrated care models for dually eligible beneficiaries? |
|  |  | What resources are available to you if you would like to refresh or obtain new knowledge pertinent to your role? |
|  |  | Are there any additional resources that would be helpful to have? |
|  |  | What do you consider your greatest success as a SHIP counselor? |
|  |  | Conversely, what do you consider your greatest challenge? |
|  | How do beneficiaries most often first connect with your SHIP program? (e.g., web site, walk in, phone/virtual, referral from another organization or state agency)? |  |
| **Counseling Content** | What topics are most commonly discussed in counseling sessions? | What topics are most commonly discussed in counseling sessions? |
|  | Are there any guidelines or tools used by counselors to help beneficiaries with their plan choice? | What guidelines or tools do you use to help beneficiaries with their plan choice? |
|  | What broader structures are in place to ensure that counselors are providing accurate and comprehensive counseling? |  |
|  | Are there any topics or questions that are particularly difficult for counselors to address or for beneficiaries to understand? | Are there any topics or questions that are particularly difficult for you to address? |
|  |  | Are there any topics or questions that are particularly difficult for beneficiaries to understand? |
|  | How often do counselors encounter dually eligible or other low-income beneficiaries? | How often do you encounter dually eligible or other low-income beneficiaries? |
|  | How often do counselors receive questions about Medicaid eligibility or other means-tested programs (i.e., Medicare Savings Programs)? | How often do you receive questions about Medicaid eligibility or other means-tested programs (i.e., Medicare Savings Programs)? |
|  | Could you talk about any counseling provided to dually eligible beneficiaries about integrated care models? | How often do you receive questions about integrated care plans such as fully integrated dually eligible special needs plans or Medicare – Medicaid plans? |
